# Identification of renal ischemia reperfusion injury-characteristic genes, pathways and immunological micro-environment features through bioinformatics approaches

**DOI:** 10.18632/aging.205471

**Published:** 2024-02-06

**Authors:** Xinghua Lv, Qian Fan, Xuanjie Li, Peng Li, Zhanhai Wan, Xuena Han, Hao Wang, Xiaoxia Wang, Lin Wu, Bin Huo, Li Yang, Gen Chen, Yan Zhang

**Affiliations:** 1 Department of Anesthesiology, First Hospital of Lanzhou University, Lanzhou, Gansu, China; 2 The First Clinical Medical College of Lanzhou University, Lanzhou, Gansu Province, China; 3 Tianjin Eye Hospital, Tianjin Key Lab of Ophthalmology and Visual Science, Tianjin Eye Institute, Nankai University Affiliated Eye Hospital, Nankai University Eye Institute, Nankai University, Clinical College of Ophthalmology, Tianjin Medical University, Tianjin, China; 4 Department of Microbiology, School of Basic Medical Sciences, Guilin Medical University, Guilin, Guangxi Zhuang Autonomous Region, China; 5 Lanzhou First People's Hospital, Lanzhou, Gansu, China

**Keywords:** ischemia reperfusion injury, gene set variation analysis, weighted gene coexpression network analysis, MiRNAs, macrophage

## Abstract

Background: Biomarkers and pathways associated with renal ischemia reperfusion injury (IRI) had not been well unveiled. This study was intended to investigate and summarize the regulatory networks for related hub genes. Besides, the immunological micro-environment features were evaluated and the correlations between immune cells and hub genes were also explored.

Methods: GSE98622 containing mouse samples with multiple IRI stages and controls was collected from the GEO database. Differentially expressed genes (DEGs) were recognized by the R package limma, and the GO and KEGG analyses were conducted by DAVID. Gene set variation analysis (GSVA) and weighted gene coexpression network analysis (WGCNA) had been implemented to uncover changed pathways and gene modules related to IRI. Besides the known pathways such as apoptosis pathway, metabolic pathway, and cell cycle pathways, some novel pathways were also discovered to be critical in IRI. A series of novel genes associated with IRI was also dug out. An IRI mouse model was constructed to validate the results.

Results: The well-known IRI marker genes (Kim1 and Lcn2) and novel hub genes (Hbegf, Serpine2, Apbb1ip, Trip13, Atf3, and Ncaph) had been proved by the quantitative real-time polymerase chain reaction (qRT-PCR). Thereafter, miRNAs targeted to the dysregulated genes were predicted and the miRNA-target network was constructed. Furthermore, the immune infiltration for these samples was predicted and the results showed that macrophages infiltrated to the injured kidney to affect the tissue repair or fibrosis. Hub genes were significantly positively or negatively correlated with the macrophage abundance indicating they played a crucial role in macrophage infiltration.

Conclusions: Consequently, the pathways, hub genes, miRNAs, and the immune microenvironment may explain the mechanism of IRI and might be the potential targets for IRI treatments.

## INTRODUCTION

**Figure 1:**
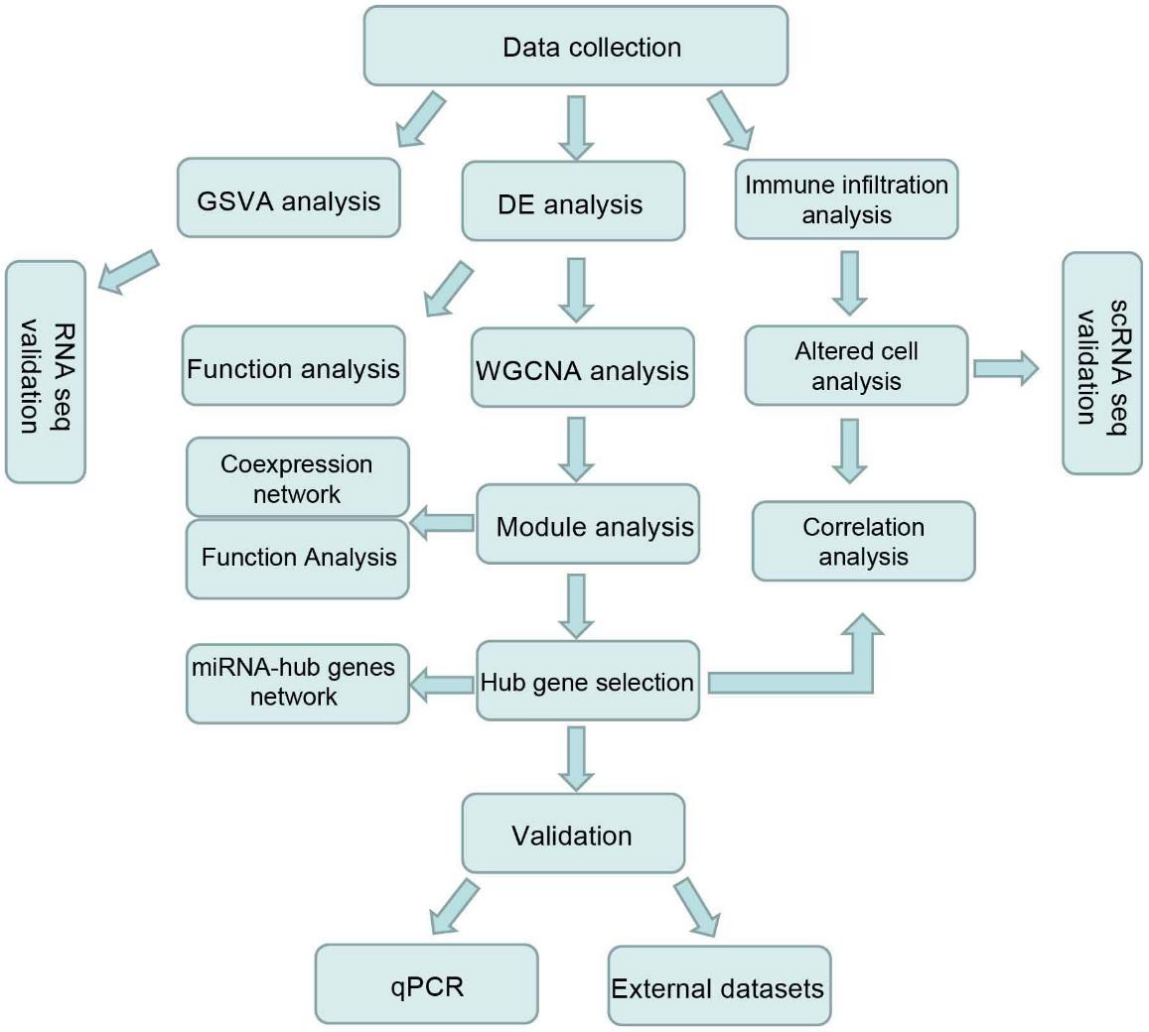
Workflow chart of this study.

Renal ischemia reperfusion injury (IRI) referred to the condition in which blood flow was interrupted and its recovery led to increased renal insufficiency and tissue damage. It was also the most common cause of acute kidney injury (AKI) resulting in death in patients with renal disease, with high morbidity and mortality [1]. As a complex disease, numerous changes in molecular characteristics happened during IRI. At present, the specific regulatory mechanism of renal IRI is not completely clear, and effective methods to prevent or treat renal IRI are rare [2]. Interpreting the concrete mechanisms of IRI may pave the way for an individualized therapeutic approach. Whole genomic analysis lays the foundation for investigating complex diseases with high efficiency. A host of studies emerged focusing on prognostic or therapeutic markers through differential expression analysis. Besides, gene correlation networks have been widely constructed to extensively understand the mechanism underlying the high-throughput sequencing data [3]. Weighted gene coexpression network analysis (WGCNA) was a common gene correlation analysis method [4], which supplied complete interpretation into the detail of molecular interactions [5–8]. In this study, RNA sequencing datasets referred to kidney IRI were collected from the Gene Expression Omnibus (GEO) database. Furthermore, differentially expressed genes (DEGs) were dug out by comparing the samples in each time point after IRI and controls (seven comparison pairs). Then, seven lists of DEGs were employed to identify gene correlation modules. The gene functional enrichment and the module trait association analysis revealed that the four modules played pivotal roles in each IRI stage and hub genes were also identified. It played important roles for immune cells and was reported to be crucial in kidney injury [9–12]. So immune cell abundances were further evaluated and the correlation between immune cells and hub genes was also calculated. Finally, the hub genes were validated by quantitative real-time polymerase chain reaction (qRT-PCR), showing potential prognostic and therapeutic targets in renal IRI. The workflow diagram of the present study was shown in Figure 1.

## MATERIALS AND METHODS

### Data collection

The IRI RNA-Seq data and the corresponding clinical data were collected from the GEO database with accession numbers GSE98622, GSE182793, and GSE139107 (Table 1). The analyses were based on GSE98622 which included six controls and 21 disease samples with seven different IRI stages. The datasets of GSE182793 and GSE139107 were set as external validations. GSE182793 included four controls and eight disease samples of IRI after 24 hours. While GES139107 was a single-cell RNA-seq dataset including control and five IRI stages with three replicates in each group. Then GSE139107 was integrated into the pseudo-bulk RNA-seq data using the R package Seurat [13] for the following analysis.

**Table 1:** Primers used in this study.

**Gene symbol**	**Primer sequence**
Kim1	Forward: TACGGCTCTCTCCTAACTGGT
	Reverse: ACCACCCCCTTTACTTCCAC
Lcn2	Forward: ACGGACTACAACCAGTTCGC
	Reverse: CCACACTCACCACCCATTCA
Hbegf	Forward: ACGCTGGGTCCTATTTGCTC
	Reverse: CGGAACACGAACGGTAGACA
Atf3	Forward:GGAAGAGCTGAGATTCGCCA
	Reverse: CTCATCTTCTTCAGGGGCCG
Apbb1ip	Forward: GCCAACCACTCATCTCTGCT
	Reverse: CCATCTTGACTGCTGGGAGG
Ncaph	Forward: TCATCTGGCCTCCCCTAACA
	Reverse: GCATCCACACGGACAGCATA
Serpine2	Forward: CACGCAAAGCCAAGACGA
	Reverse:GTCACTTAACTGCTGCTATGAACC
Trip13	Forward:GCATCTATGTAAAGCCCCATCC
	Reverse:TCTAGCCTGAGCAAAGAATCCA
GAPDH	Forward:TGTGTCCGTCGTGGATCTGA
	Reverse:TTGCTGTTGAAGTCGCAGGAG

### Screening of DEGs

Limma package [14] was performed to identify DEGs for the IRI stage vs. the control group for GSE98622 after transforming the FPKM to TPM. DEseq2 [15] was used to identify DEGs for GSE182793 with count data as input. Genes with log2|fold change| > 1, and adjusted p-value < 0.01 from multiple testing for p-values by the Benjamini–Hochberg adjustment method were identified to be significantly differentially expressed [16].

### Weighted gene coexpression network construction

WGCNA was used to identify clusters of genes with similar expression profiles using Pearson’s correlation coefficients. A predefined β value was calculated and adopted to build the network topological overlap measure matrix (TOM) which was used to measure the connectivity of the pair of genes [17]. And then hierarchical clustering of average linkage was applied to cut the gene coexpression network to coexpression modules on the basis of the network topology overlap [18]. The IRI stage representative modules were screened out using the relationships between the modules and external clinical traits (Spearman correlation between the eigengene and sample stages) and the correlations between gene significance (GS) and module membership (MM) values. Stage specific modules were filtered by correlation > 0.65 and p-value < 0.01 between the module eigengene (ME) and the IRI stages. Hub genes were defined by GS > 0.2 and MM > 0.9 in a specific module.

### Functional enrichment analysis for DEGs

The Database for Annotation, Visualization, and Integrated Discovery (DAVID) web tool was utilised to carry out the GO term and KEGG pathway enrichment analysis [19]. A BH adjusted p-value < 0.05 was considered significant.

### Gene set variation analysis

GSVA was a nonparametric, unsupervised method used to calculate the enrichment score of a specific gene set in each sample [20]. To study the biological variation between each IRI stage and the control group, we analyzed the differential expression of dysregulated pathways using the R package GSVA (v1.40.1) [20]. Hallmark pathways for the mouse in the MSigDB database was used in this analysis (https://www.gseamsigdb.org/gsea/msigdb) [21, 22].

### Immune cell infiltration estimation

The immune cell infiltration for GSE98622 was estimated by ImmunCellAI-mouse [23] which can predict 36 immune cell types. There were three layers of immune cells and layer one was used in the present study including seven types of cells. That was B cell, NK cell, T cell, Macrophage, Dendritic cell, Monocyte, and Granulocyte.

### Single cell RNA-seq analysis

The R package “Seurat” [13] was utilized for scRNA-seq data preprocessing and analysis. All the samples were merged into an integrated dataset using R package harmony [24, 25]. Principal Component Analysis (PCA) on the highly variable genes were performed, utilizing the top 30 principal components for subsequent analyses. Cell type annotation was obtained from Kirita et al. [26].

### Experimental animals

Twelve 7-week-old male C57BL/6 mice were subdivided into the sham group (S group, n=3), renal I/R groups of 4 hours (group I/R 4h, n=3), renal I/R groups of 24 hours (group I/R 24h, n=3) and renal I/R groups of 7 days (group I/R 7d, n=3). All these experiments were guided by the requirements of the Lanzhou University Animal Care and Use Committee.

### Renal I/R model

Pentobarbital sodium (70mg/kg) was intraperitoneally injected as an anesthetic. No occlusion of the renal pedicles was chosen in the S group. Renal IRI followed by reperfusion under body temperature (37° C) was executed in other groups. Corresponding mice tissue samples were harvested at 4h, 24h, and 7d after reperfusion from each group.

### Immunohistochemistry and qRT-PCR

10% formalin was used for renal tissue fixation for 24 h and then the tissue samples were embedded in paraffin. All samples were stained with hemotoxylin-eosin and compared histological changes under the light microscope. TRIzol reagent (Thermo Fisher Scientific, USA) and miRNeasy Mini Kit (Qiagen, Shanghai, China) following the instructions were used for RNA extraction. Kim1, Lcn2, Hbegf, Serpine2, Apbb1ip, Trip13, Atf3, and Ncaph were tested through reverse transcription and amplification (Table 1).

### Statistical analysis

R 4.1.2 was implemented to analyze the datasets. Data from different groups were compared using the Wilcoxon test or Student’s t test. The p-value < 0.05 was considered statistically significant unless otherwise specified.

## RESULTS

### Data description

**Figure 2:**
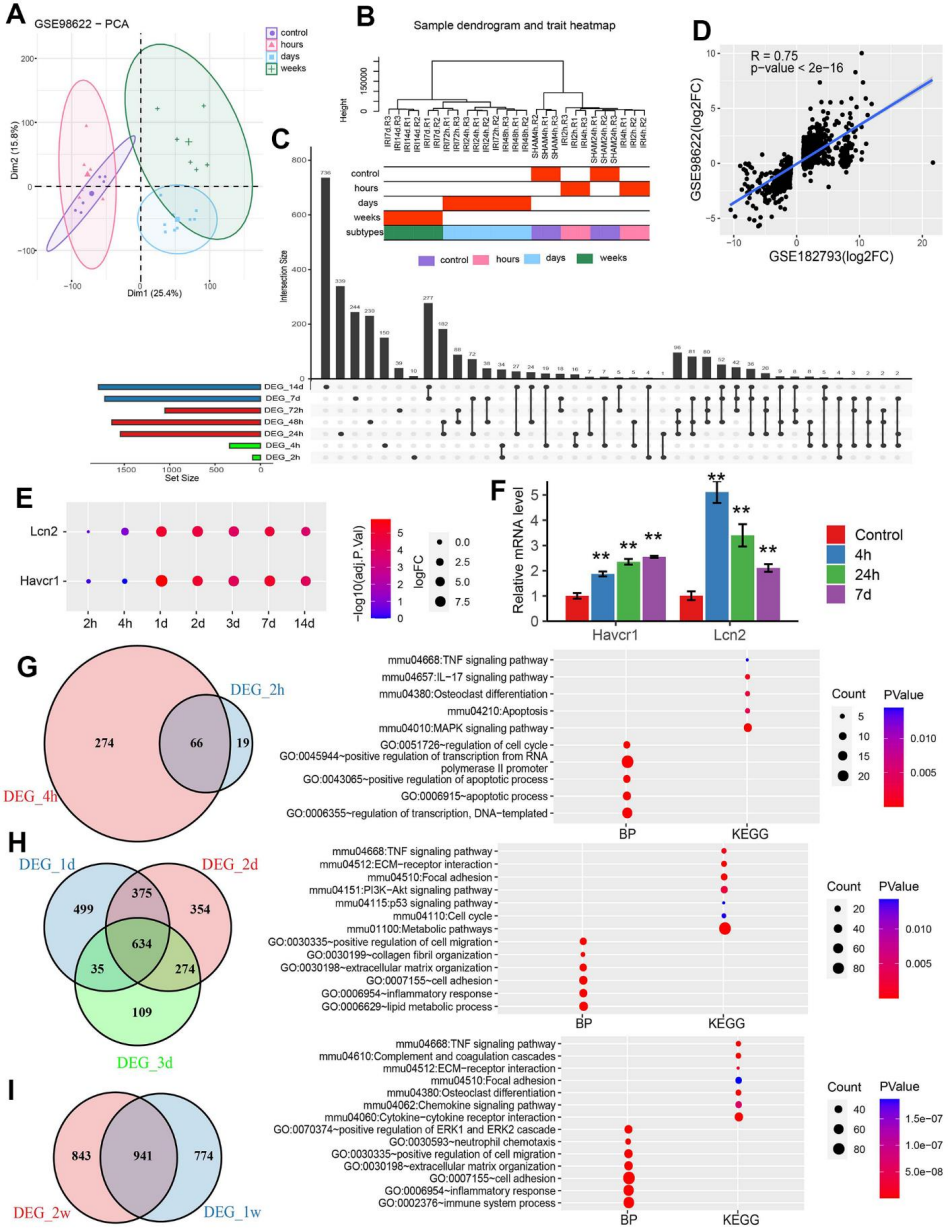
**Differential analysis for GESE98622.** (**A**) PCA plot of GSE98622. (**B**) Sample clustering of GSE98622. (**C**) The upset plot of DEGs was identified at each time point. (**D**) The scatter plot of DEGs in GSE98622 and GSE182793 after IRI 24h. (**E**) The dotplot of two AKI marker genes. (**F**) The qRT-PCR results of two well-known IRI related genes in the mouse IRI group and the normal group. * P<0.05, **P<0.01. (**G**) The common DEGs and functions in the hour group. (**H**) The common DEGs and functions in the day group. (**I**) The common DEGs and functions in the week group.

Three datasets related to IRI in mouse kidneys from GEO database were analyzed (Table 2). The data from GSE98622 for mouse kidneys were interpreted by analyzing its distribution in a PCA plot, which located each sample in different dimensions. Samples within the IRI stages were in close proximity (Figure 2A). Similarly, the hierarchical clustering analysis showed that the samples originated from the same group were clustered together (Figure 2B). According to the above two figures, we divided the samples into four groups/IRI stages including a group of controls, a group of hours with samples after IRI for 2 and 4 hours, a group of days with samples after IRI for one day, two days and three days, and a group of weeks including samples after IRI for one week and two weeks.

**Table 2:** The summary of the datasets collected from the GEO database.

**Reference**	**GEO series**	**Data type**	**Control**	**IRI**
Liu, Kumar et al. 2017 [24]	GSE98622	RNA-seq	6	21
Verney, Legouis et al. 2021 [25]	GSE182793	RNA-seq	4	8
Kirita, Wu et al. 2020 [26]	GSE139107	scRNA-seq	4	20

### Identification of DEGs between IRI groups and controls

The DEGs were identified between samples in each time point and control. The upset plot showed that the number of DEGs increased with the injury progress. Considerable DEGs emerged in 24h after injury (Figure 2C). There was a significant correlation between DEGs in GSE98622 and GSE182793 in 24h after injury (Figure 2D). The two clinically recognized tubular injury markers, Kim1 (Havcr1) and Lcn2 were also significantly upregulated after one day of injury (Figure 2E). qRT-PCR results also showed significant upregulation of these two genes early at 4 hours after injury (Figure 2F). Venn diagrams were plotted for each IRI group. In the group of hours including DEGs after two hours and four hours of injury, 66 common genes were identified and they were involved in the apoptosis process (Figure 2G). In the group of days with DEGs from one day, two days, and three days after IRI, 634 common DEGs were identified which were engaged in metabolic pathways (Figure 2H). A total of 941 DEGs in the group of weeks including DEGs in one week and two weeks after IRI were associated with immune responses (Figure 2I).

### Gene set variation analysis for IRI groups and controls

**Figure 3:**
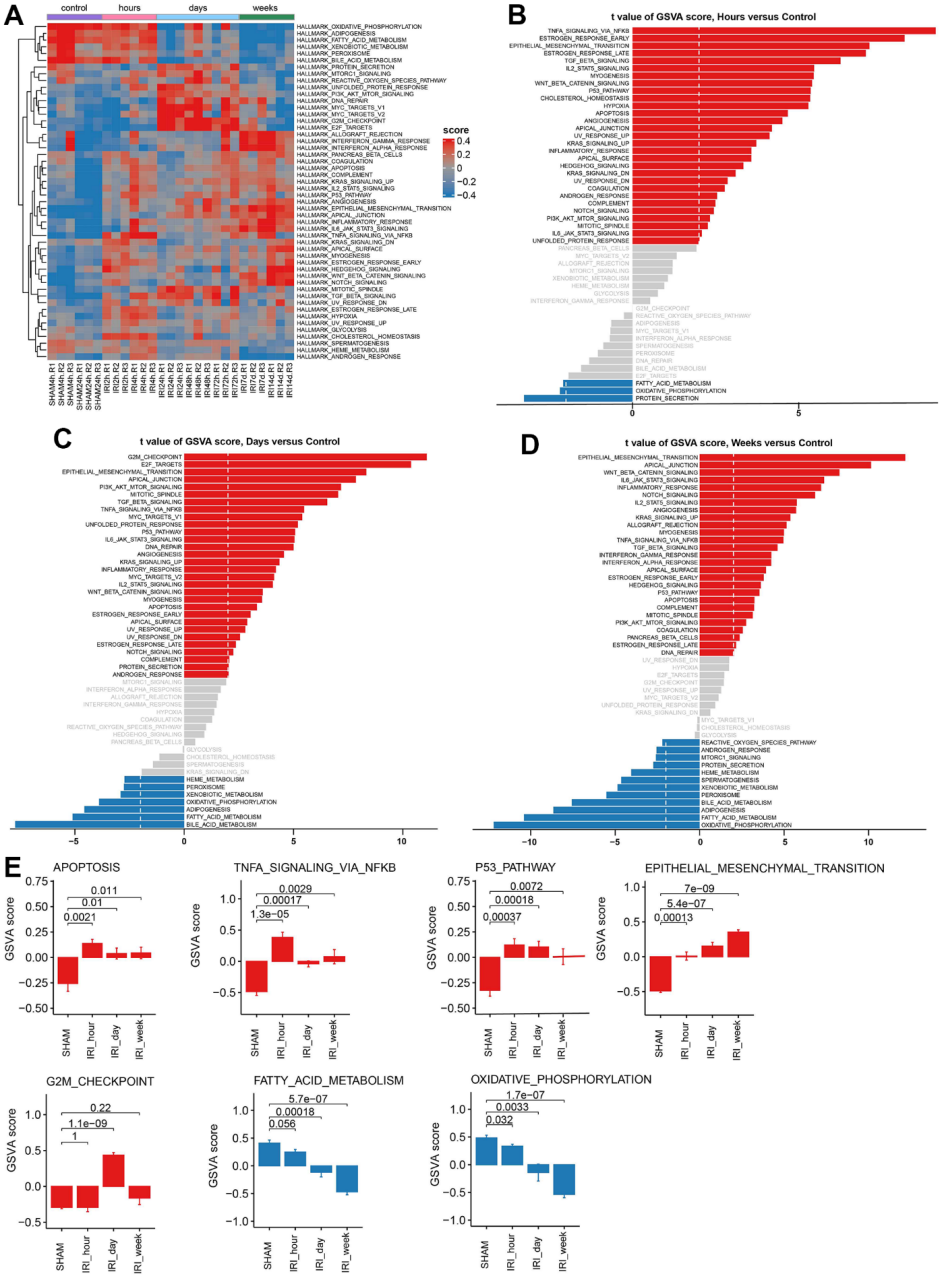
**Gene set variation analysis for hallmark pathways.** (**A**) The heatmap of GSVA results. (**B**) The barplot of GSVA score for group hours vs. Control. (**C**) The barplot of GSVA score for group days vs. Control. (**D**) The barplot of GSVA score for group weeks vs. Control. (**E**) The barplot of GSVA scores in different hallmark pathways.

GSVA analysis was performed to investigate the activated and suppressed pathways in IRI groups. The results showed that a series of pathways were changed early in the hours after IRI (Figure 3A). In detail, the TNFA signaling via the NFKB pathway, p53 pathway, and apoptosis pathway was activated in the group of hours (Figure 3B, 3E). Then the pathways related to the cell cycle were upregulated in the group of days (Figure 3C, 3E). In the group of weeks, functions of epithelial mesenchymal transition were activated (Figure 3D, 3E). The oxidative phosphorylation and the fatty acid metabolism pathways were suppressed during all the IRI stages (Figure 3E). Besides those well-known IRI associated pathways, some novel pathways were also significantly altered such as estrogen response early/late, hedgehog signaling, and cholesterol homeostasis (Figure 3A). Similar results were found in GSE182793 and GSE139107 using GSVA analysis (Supplementary Figure 1).

### Weighted gene coexpression network analysis

**Figure 4:**
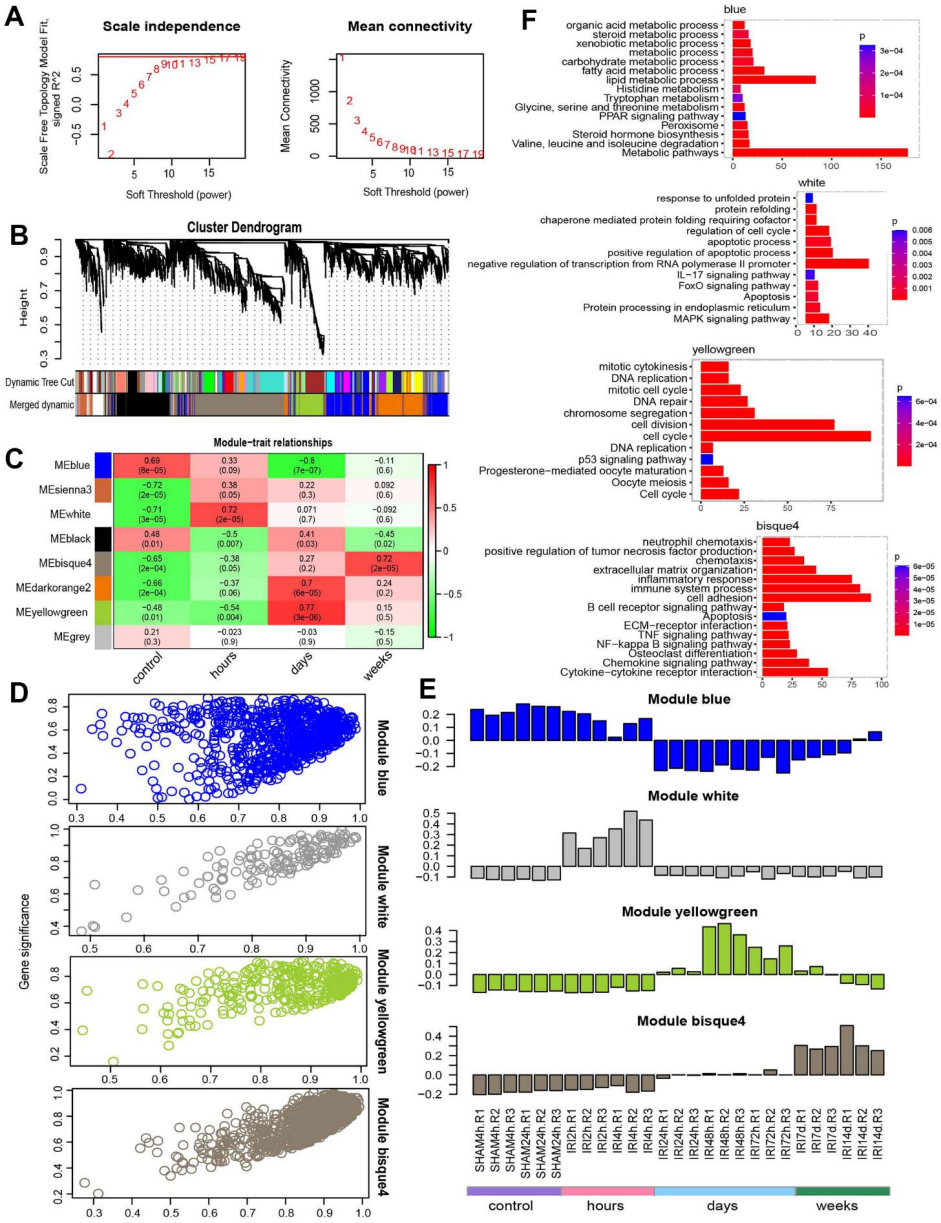
**WGCNA analysis.** (**A**) Power selection. (**B**) Dendrogram of coexpression network. (**C**) The module trait association heatmap. (**D**) Scatter plot for module membership and gene significance. (**E**) Barplot for the eigengene in each module. (**F**) The functions for each module.

**Figure 5:**
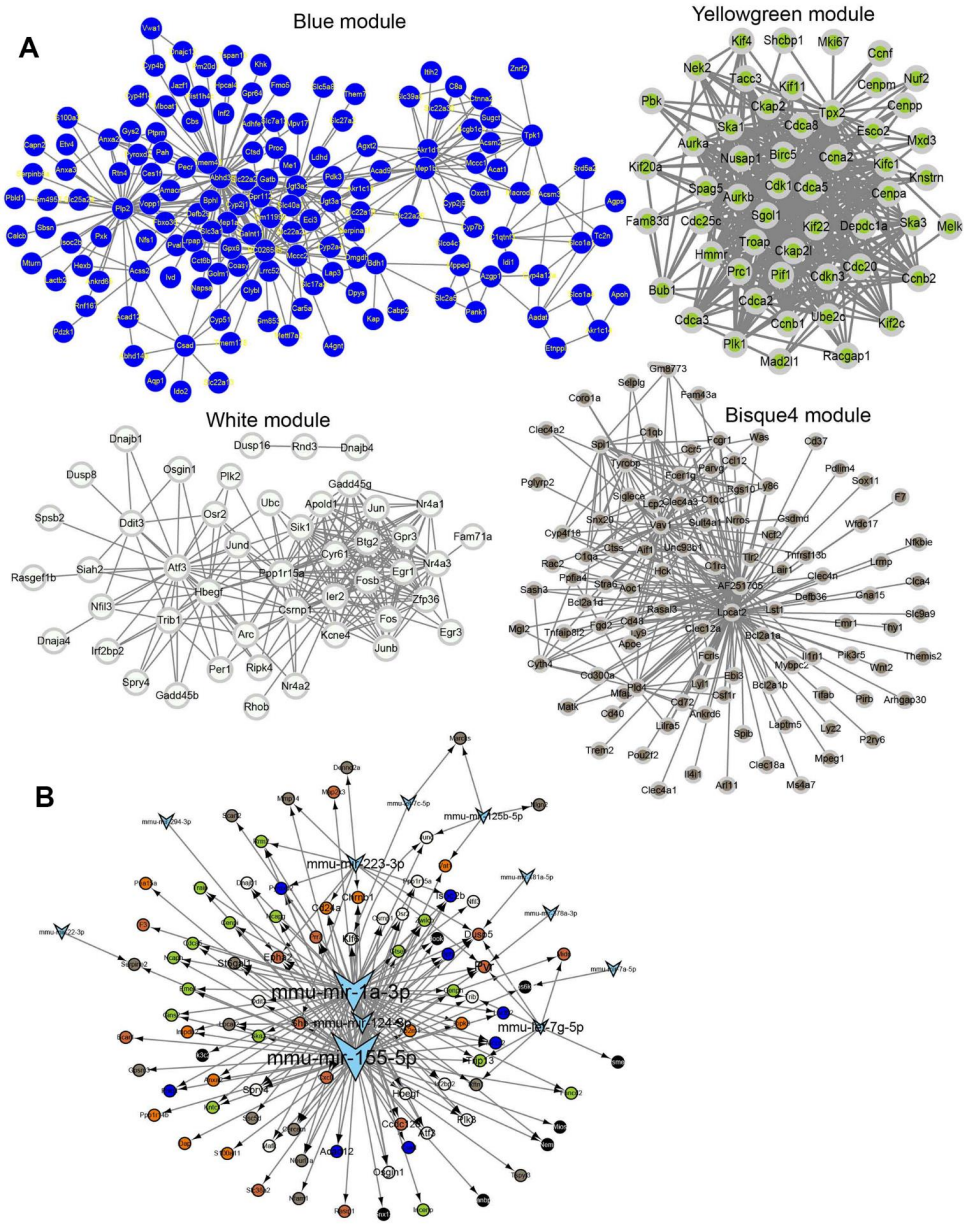
**Network for each module.** (**A**) Coexpression network for module blue, white, yellow, green and bisque4. (**B**) The regulatory network of IRI-related genes and miRNAs.

Different modules connected with different IRI stages were identified by WGCNA using the 3,691 union DEGs from the above DE analysis. The soft threshold power of the network topology was analyzed with a β value from 1 to 20. The scale independence was calculated with a threshold of 17 (Figure 4A). Next, the β = 17 was selected to generate a tree of hierarchical clustering genes (Figure 4B). Seven modules with different gene numbers were identified (Figure 4B and Table 2). Then the correlation analysis between module eigengenes and the external traits such as different IRI groups was performed. Module blue, module white, module yellowgreen, and module bisque4 were calculated with the most significant correlation to group control, hours, days, and weeks respectively (Figure 4C). The association was demonstrated between the blue, white, yellowgreen, bisque4, and the genetic significance (Figure 4D). And the bar plot of eigengene in each module was shown in Figure 4E. Gene functional enrichment was used for the coexpression modules (Table 3). Blue module genes participated in metabolic related pathways such as lipid metabolic process, fatty acid metabolism, and amino acid degradation (Figure 4F and Table 3). White module genes were significantly related to apoptosis (Figure 4F and Table 3). Genes in module yellowgreen were cell cycle related (Figure 4F and Table 3). And immune response associated functions were enriched in module bisque4 (Figure 4F and Table 3). Then the coexpression networks of the four major modules were constructed (Figure 5A). And the top five hub genes in each module were also listed in Table 3.

**Table 3:** Module description.

**Module**	**Size**	**Functions**	**p.adjust**	**Hub genes**
Blue	774	Lipid metabolic process	3.06E-20	Acss2,Csad,Mep1a,Gatb,Slc3a1
		Fatty acid metabolic process	7.99E-10	
		Metabolic pathways	1.34E-36	
Sienna3	133	TNF signaling pathway	6.81E-07	Epha2,Shb,Ccdc120,Slc38a2,Prr7
		Inflammatory response	1.13E-04	
		IL-17 signaling pathway	2.62E-04	
White	139	Positive regulation of apoptotic process	2.89E-05	Hbegf,Atf3,Trib1,Ddit3,Osr2
		Apoptotic process	1.36E-02	
		Apoptosis	1.47E-02	
Black	606	Regulation of transcription from RNA Polymerase II promoter	9.88E-17	Capn7,Mkln1,Mios,Dhx36,Pik3c2a
		DNA repair	1.05E-07	
		Cell cycle	3.39E-03	
Bisque4	953	Inflammatory response	3.43E-23	Serpine2,Apbb1ip,Cercam,Cd276,Dennd2a
		Cell adhesion	1.08E-21	
		Immune system process	2.43E-19	
Darkorange2	655	Extracellular region	4.82E-07	Chrnb1,S100a11,Tpm3,Impdh2,Ifitm10
		Translation	2.81E-05	
		Ribosome	2.37E-05	
Yellowgreen	334	Cell cycle	3.28E-67	Ncaph,Trip13,Zwilch,Gtse1,Ncapg
		DNA repair	2.50E-08	
		Cell cycle	1.18E-14	

### miRNAs prediction for the coexpression modules

The miRNAs targeting the top 20 hub genes in each module were predicted by the miRNet database. The results showed that mmu-mir-155-5p, mmu-mir-1a-3p, mmu-mir-124-3p, and mmu-mir-223-3p played important roles in regulating these hub genes (Figure 5B).

### Immune infiltration evaluation for IRI

**Figure 6:**
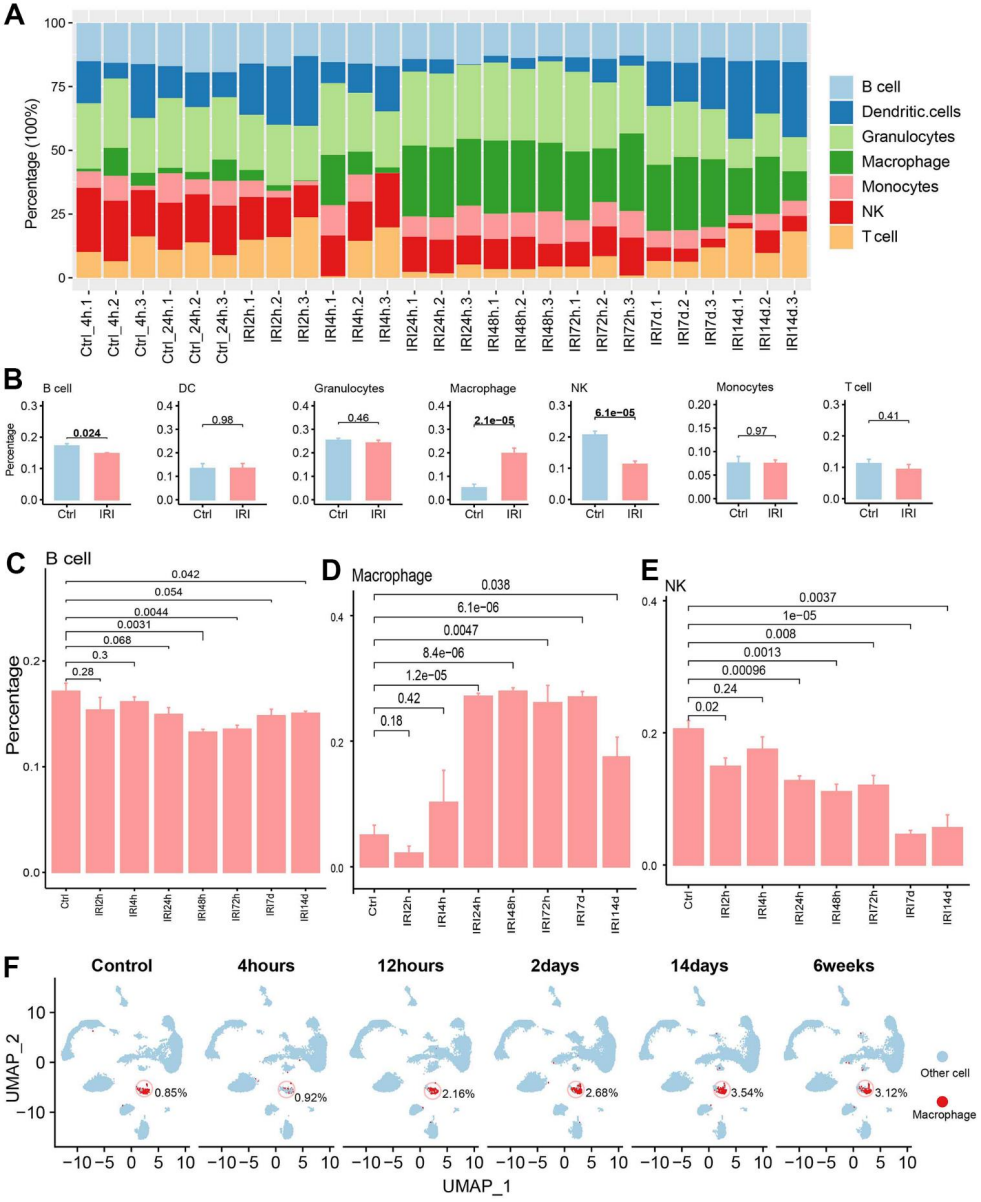
**The immune cell infiltration analysis.** (**A**) The immune cell abundance in each sample. (**B**) The immune cell abundance difference between the control and IRI groups. (**C**) The B cell abundance difference between the control and each IRI group. (**D**) The macrophage abundance difference between the control and each IRI group. (**E**) The T cell abundance difference between the control and each IRI group. (**F**) The abundance of macrophages across different IRI stages in GSE139107 using UMAP plot.

The immune cell abundance in IRI samples and controls was evaluated (Figure 6A). And there were significant differences for the immune cells between group IRI and the control (Figure 6B). B cells and NK cells decreased in the IRI group, while macrophage increased across the IRI stages (Figure 6C–6E). Then the single cell dataset of GSE139107 also showed that the macrophage increased after IRI and kept the increase to 6 weeks after IRI (Figure 6F).

### The correlation between hub genes and macrophage infiltration

**Figure 7:**
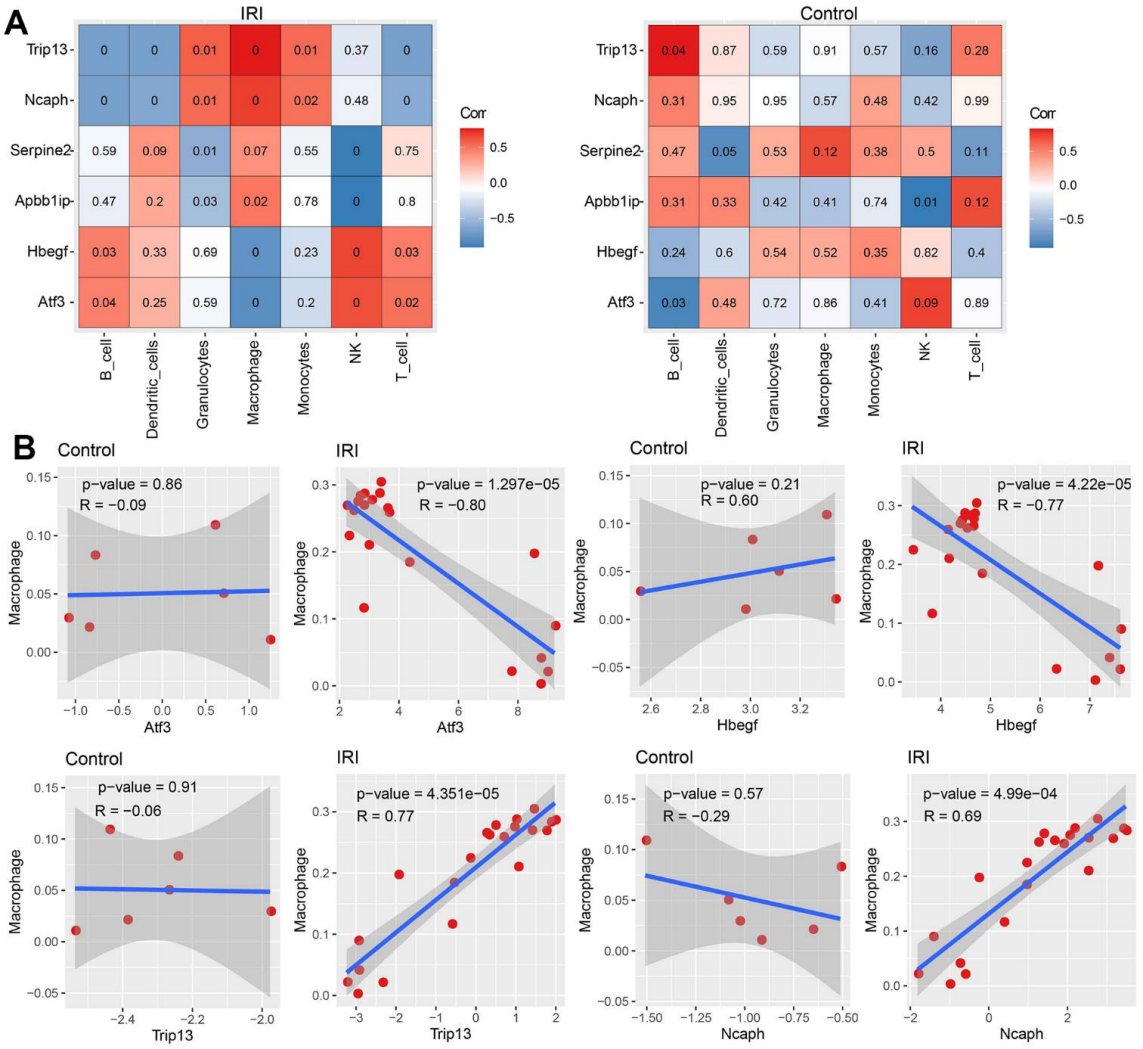
**The correlation between immune cell abundance and hub genes expression.** (**A**) The correlation between immune cell abundance and hub genes expression in IRI and control groups. (**B**) The correlation between macrophage abundance and each hub gene expression in each group.

Pearson correlation coefficients were calculated between each immune cell and each hub gene under control or IRI condition. The results showed significant correlations between immune cells and hub genes in IRI, while no or less correlation in the control group (Figure 7A). Hub genes of Atf3 and Hbegf negatively correlated with macrophage while Trip13 and Ncaph positively correlated with macrophage significantly in IRI (Figure 7B).

### Immunohistochemistry for IRI mouse model and qRT-PCR for hub genes

**Figure 8:**
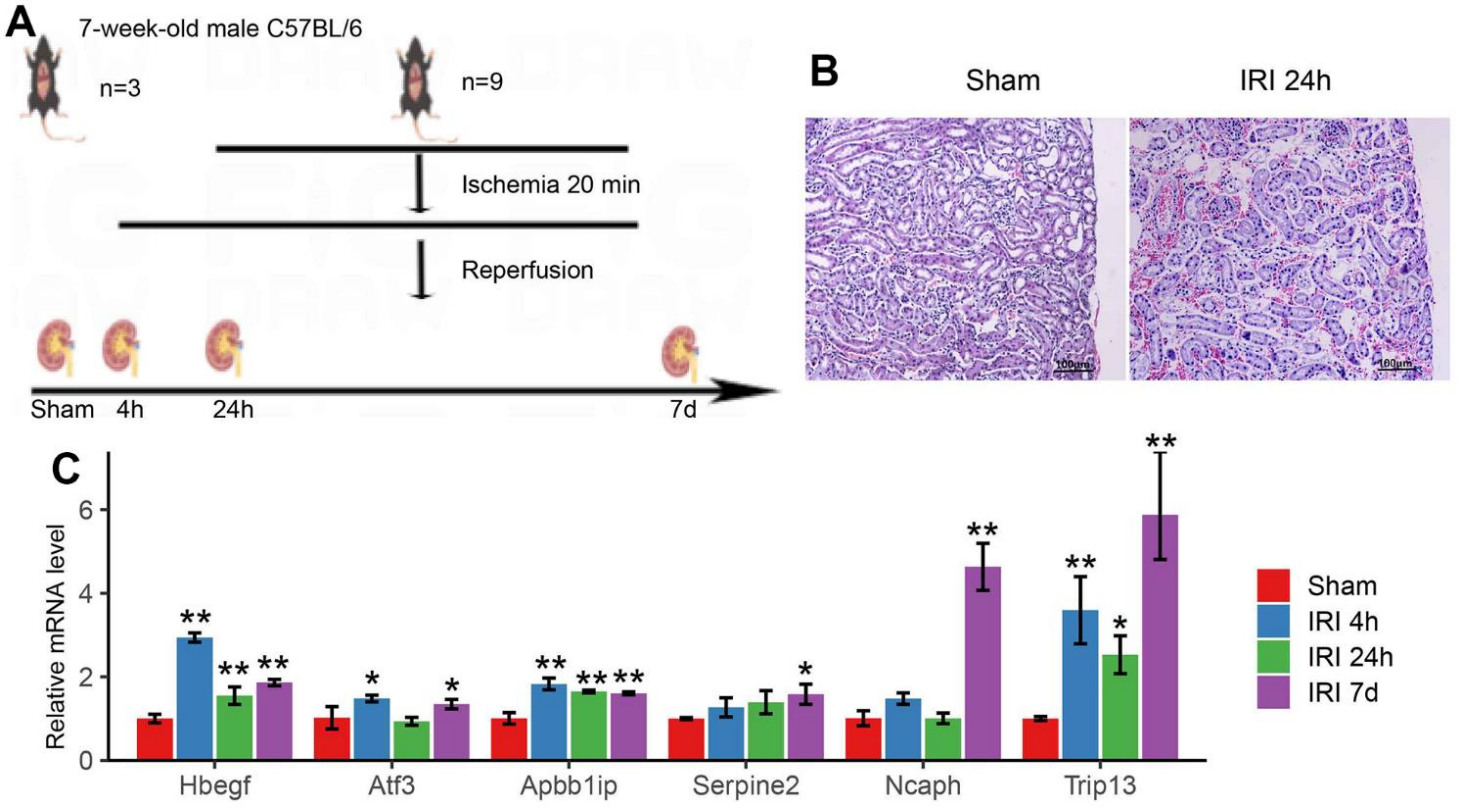
(**A**) The schematic graph of the mouse IRI model. (**B**) Pathological changes of renal tissue in 24h of IRI. (**C**) The qRT-PCR results of hub genes in the mouse IRI group and the normal group. * p<0.05, **p<0.01.

In order to evaluate the expression level of hub genes, the IRI mouse model was constructed (Figure 8A). Histological samples were obtained to analyze the alterations in the mouse renal tissue structure after IRI (24 h). It was intact and clear for the renal tissue structure with closely arranged and well-defined renal tubules, and no inflammatory cell infiltration in the sham group. While in the group of IRI after 24h, severely damaged tissue was observed with loose tissue arrangement and enlarged tissue gap, as well as hyperemia, swelling, and inflammatory cell infiltration (Figure 8B). qRT-PCR was used to confirm the expression of hub genes in IRI in the mouse model. The results indicated that the hub genes were up-regulated in IRI (P<0.01) (Figure 8C). For the hub genes of Hbegf and Atf3, the qPCR showed upregulation in 4h after IRI which was in accord with the results in WGCNA. While Ncaph and Trip13 significantly increased in 7 days after IRI, which worked as the marker genes in the late stage of IRI.

## DISCUSSION

This study investigated gene transcriptional datasets to find potential pathways and biomarkers in different IRI stages and confirmed the findings by the external datasets and qRT-PCR experiments. The differential expression analysis showed the expression level of Kim1 (Havcr1) and Lcn2 were increased in samples after 24h of IRI and kept the elevation to 7 days. Kim1 participated in renal tubular injury, inflammation, and fibrosis, preventing AKI to chronic kidney diseases [27]. Lcn2 was also investigated as an injury marker for IRI. GSVA was performed to investigate the activated pathways in IRI stages. The results showed that different pathways were activated across the IRI progress. In the early stage of IRI (hours), the apoptosis pathway was activated which meant that the IRI could induce apoptosis at a very early stage [28]. As the injury progressed, cell cycle related functions were activated [29]. It was reported that the wound healing was determined by the cell cycle arrest in the epithelial after injury leading to either normal cell proliferation or fibrosis. Thus, cell cycle arrest might be a potential therapeutic strategy in organ fibrosis after injury [30]. In the present study, we found that the cell cycle related pathways were activated, so the inhibitors targeting the cell cycle pathways may prevent kidney fibrosis. Then the functions of the immune response were activated. It was well-known that kidney IRI was associated with the adaptive and innate immune system and the activation of the immune system was crucial in IRI [31, 32]. Besides those above known pathways, some novel pathways also were found to be associated with IRI such as estrogen response early/late, hedgehog signaling, and cholesterol homeostasis. These results revealed that multiple pathways participated in the course of IRI.

A total of 3,691 union DEGs were used to construct the weighted gene correlation network and then were divided into different gene correlation modules associated with IRI stages. The modules named blue, white, bisque4, and yellowgreen which were found to be highly associated with each IRI stage were dissected. Then in order to uncover the functions each module played during IRI, the module functional enrichment analysis was conducted. Blue groups with decreasing gene expression in IRI groups are involved in metabolic related pathways meaning that the metabolism was suppressed after IRI. The IRI could induce apoptosis early at 2h of injury indicated by the module white. The functions of genes in module yellowgreen were cell cycle and then a series of immune response associated functions were activated in module bisque4. Additionally, hub genes such as Atf3, Hbegf, Serpine2, Apbb1ip, Trip13, and Ncaph with high connectivity were screened out from each module and validated by qRT-PCR using a mouse model. Because of the importance of immune cells in IRI, we predicted the immune infiltration for injured samples. Huen et al., reported that macrophages became activated and increased from the damaged microenvironment to promote tissue injury or repair [11]. Macrophage accumulation induced the occurrence of kidney injury which was alleviated by the removal of renal macrophages [33]. Whereas macrophages protected the kidney injury by secreting growth factors to promote tissue repair and remodeling [34]. The role of macrophage was complicated and bidirectional in the kidney injury process. There were numerous genes to regulate macrophage infiltration in injured tissue and positive or negative correlations between hub genes and macrophages. The hub genes are involved in macrophage infiltration in damaged tissue. Activating transcription factor 3 (ATF3) belonged to the ATF/CREB family of the basic-region leucine zipper (bZIP) family. ATF3 played a protective role in alleviating the inflammatory response in tubular cell death and nephrotoxicity induced by IRI. ATF3 indicated the occurrence of AKI which was associated with strong inflammation with increasing secretion of cytokines, such as IL-6, IL-12, and IFNγ [35]. A higher expression level of ATF3 significantly inhibited the infiltration level of macrophages to be beneficial for patients with tumors [36]. A significantly negative correlation between ATF3 and macrophages was found in our study, indicating the potential of ATF3 to be a therapeutic target by regulating the infiltration of macrophages. Heparin binding EGF-like growth factor (HBEGF) was a biologically active protein that acted as an intestinal cytoprotective agent. James et al. reported that HBEGF alleviated acute lung injury in IRI mice and worked as a systemic anti-inflammatory agent preventing systemic inflammatory response syndrome after intestinal injury [37]. And it was also significantly negatively correlated with macrophage and might inhibit the infiltration of macrophage. Thyroid receptor interacting protein 13 (TRIP13) was a common type of renal stressor and critical for the repair of the tubular epithelial cell in IRI. TRIP13 insufficiency increased the probability of damaged tubular epithelial cells progressing toward apoptotic cell death [38]. In our study, there was a significant positive correlation between TRIP13 and macrophage infiltration unveiling the functions of TRIP13 in regulating the immune response in IRI. Furthermore, other hub genes may play important roles in IRI and need to be validated in the future.

MiRNAs had been proven to play protective roles in IRI as critical regulators of cellular processes such as differentiation, proliferation, and apoptosis. Utilizing the miRNet, we predicted the potential miRNAs for the hub genes in the coexpression network. A series of miRNAs were discovered in regulating the hub genes’ network including mir-155-5p, mir-1a-3p, mir-124-3p, and mir-223-3p. Wu et al. reported that miR-155/FoxO3a/ARC led to renal pyroptosis under IRI conditions and verified that miR-155 played a crucial role in the pathogenesis of renal tubular cell pyroptosis, suggesting that miR-155 might be a potential therapeutic target in the treatment of ischemic renal diseases [39]. Previous study reported that pyroptosis of renal cells in IRI was inhibited by miR-155-5p/DDX3X/NLRP3/caspase-1 pathway, indicating that miR-155-5p played a critical role in the pathogenesis of renal tubular cell pyroptosis as a potential therapeutic target [40]. Additionally, apoptosis occurred in all the IRI stages, which was mediated by endoplasmic reticulum stress (ERS). Ding et al. reported that miR-124 played an important role in ERS in renal IRI. They found that miR-124 bound to IRE-1α as a negative regulator of ERS, and then conferred its protective effect, which demonstrated the regulatory mechanism of miR-124 in renal IRI and provided new ideas and methods for the prevention and treatment of renal IRI [41]. Although there were no reports about miR-1a-3p in IRI, we speculated that it might also play a similar role in IRI.

## CONCLUSIONS

In summary, this study revealed the regulatory networks of related hub genes of renal IRI. This study successfully identified a host of pathways, miRNAs, hub genes as well as the immune microenvironment to explain the mechanism of IRI. Accumulated evidence indicated the novel, potential, and promising targets for future IRI treatments.

## Supplementary Material

Supplementary Figure 1

## References

[1] Platt E, Klootwijk E, Salama A, Davidson B, Robertson F. Literature review of the mechanisms of acute kidney injury secondary to acute liver injury. World J Nephrol. 2022; 11:13–29. 10.5527/wjn.v11.i1.13 35117976 PMC8790308

[2] Snoeijs MG, van Heurn LW, Buurman WA. Biological modulation of renal ischemia-reperfusion injury. Curr Opin Organ Transplant. 2010; 15:190–9. 10.1097/MOT.0b013e32833593eb 20009928

[3] Pan Z, Yang Y, Cao R, Qiu Y, Li S, Zhao Y, Chang S, Chen S, Chen Z, Zhang W, Zhao D. Identification and Verification of Potential Biomarkers in Renal Ischemia-Reperfusion Injury by Integrated Bioinformatic Analysis. Biomed Res Int. 2023; 2023:7629782. 10.1155/2023/7629782 36778059 PMC9911259

[4] Langfelder P, Horvath S. WGCNA: an R package for weighted correlation network analysis. BMC Bioinformatics. 2008; 9:559. 10.1186/1471-2105-9-559 19114008 PMC2631488

[5] Yuan Y, Li N, Fu M, Ye M. Identification of Critical Modules and Biomarkers of Ulcerative Colitis by Using WGCNA. J Inflamm Res. 2023; 16:1611–28. 10.2147/JIR.S402715 37092131 PMC10120594

[6] Chen D, Yi R, Hong W, Wang K, Chen Y. Anoikis resistance of small airway epithelium is involved in the progression of chronic obstructive pulmonary disease. Front Immunol. 2023; 14:1155478. 10.3389/fimmu.2023.1155478 37090717 PMC10113535

[7] Zhang M, Wang X, Chen W, Liu W, Xin J, Yang D, Zhang Z, Zheng X. Integrated bioinformatics analysis for identifying key genes and pathways in female and male patients with dilated cardiomyopathy. Sci Rep. 2023; 13:8977. 10.1038/s41598-023-36117-0 37268658 PMC10238547

[8] Zhu E, Shu X, Xu Z, Peng Y, Xiang Y, Liu Y, Guan H, Zhong M, Li J, Zhang LZ, Nie R, Zheng Z. Screening of immune-related secretory proteins linking chronic kidney disease with calcific aortic valve disease based on comprehensive bioinformatics analysis and machine learning. J Transl Med. 2023; 21:359. 10.1186/s12967-023-04171-x 37264340 PMC10234004

[9] Ma R, Jiang W, Li Z, Sun Y, Wei Z. Intrarenal macrophage infiltration induced by T cells is associated with podocyte injury in lupus nephritis patients. Lupus. 2016; 25:1577–86. 10.1177/0961203316646861 27147620

[10] Williams TM, Little MH, Ricardo SD. Macrophages in renal development, injury, and repair. Semin Nephrol. 2010; 30:255–67. 10.1016/j.semnephrol.2010.03.011 20620670

[11] Huen SC, Cantley LG. Macrophages in Renal Injury and Repair. Annu Rev Physiol. 2017; 79:449–69. 10.1146/annurev-physiol-022516-034219 28192060

[12] Chen H, Liu N, Zhuang S. Macrophages in Renal Injury, Repair, Fibrosis Following Acute Kidney Injury and Targeted Therapy. Front Immunol. 2022; 13:934299. 10.3389/fimmu.2022.934299 35911736 PMC9326079

[13] Butler A, Hoffman P, Smibert P, Papalexi E, Satija R. Integrating single-cell transcriptomic data across different conditions, technologies, and species. Nat Biotechnol. 2018; 36:411–20. 10.1038/nbt.4096 29608179 PMC6700744

[14] Ritchie ME, Phipson B, Wu D, Hu Y, Law CW, Shi W, Smyth GK. limma powers differential expression analyses for RNA-sequencing and microarray studies. Nucleic Acids Res. 2015; 43:e47. 10.1093/nar/gkv007 25605792 PMC4402510

[15] Love MI, Huber W, Anders S. Moderated estimation of fold change and dispersion for RNA-seq data with DESeq2. Genome Biol. 2014; 15:550. 10.1186/s13059-014-0550-8 25516281 PMC4302049

[16] Li J, Witten DM, Johnstone IM, Tibshirani R. Normalization, testing, and false discovery rate estimation for RNA-sequencing data. Biostatistics. 2012; 13:523–38. 10.1093/biostatistics/kxr031 22003245 PMC3372940

[17] Yip AM, Horvath S. Gene network interconnectedness and the generalized topological overlap measure. BMC Bioinformatics. 2007; 8:22. 10.1186/1471-2105-8-22 17250769 PMC1797055

[18] Ravasz E, Somera AL, Mongru DA, Oltvai ZN, Barabási AL. Hierarchical organization of modularity in metabolic networks. Science. 2002; 297:1551–5. 10.1126/science.1073374 12202830

[19] Dennis G Jr, Sherman BT, Hosack DA, Yang J, Gao W, Lane HC, Lempicki RA. DAVID: Database for Annotation, Visualization, and Integrated Discovery. Genome Biol. 2003; 4:P3. 10.1186/gb-2003-4-5-p312734009

[20] Hänzelmann S, Castelo R, Guinney J. GSVA: gene set variation analysis for microarray and RNA-seq data. BMC Bioinformatics. 2013; 14:7. 10.1186/1471-2105-14-7 23323831 PMC3618321

[21] Liberzon A, Birger C, Thorvaldsdóttir H, Ghandi M, Mesirov JP, Tamayo P. The Molecular Signatures Database (MSigDB) hallmark gene set collection. Cell Syst. 2015; 1:417–25. 10.1016/j.cels.2015.12.004 26771021 PMC4707969

[22] Liberzon A, Subramanian A, Pinchback R, Thorvaldsdóttir H, Tamayo P, Mesirov JP. Molecular signatures database (MSigDB) 3.0. Bioinformatics. 2011; 27:1739–40. 10.1093/bioinformatics/btr260 21546393 PMC3106198

[23] Miao YR, Xia M, Luo M, Luo T, Yang M, Guo AY. ImmuCellAI-mouse: a tool for comprehensive prediction of mouse immune cell abundance and immune microenvironment depiction. Bioinformatics. 2022; 38:785–91. 10.1093/bioinformatics/btab711 34636837

[24] Liu J, Kumar S, Dolzhenko E, Alvarado GF, Guo J, Lu C, Chen Y, Li M, Dessing MC, Parvez RK, Cippà PE, Krautzberger AM, Saribekyan G, et al. Molecular characterization of the transition from acute to chronic kidney injury following ischemia/reperfusion. JCI Insight. 2017; 2:e94716. 10.1172/jci.insight.94716 28931758 PMC5612583

[25] Verney C, Legouis D, Placier S, Migeon T, Bonnin P, Buob D, Hadchouel J, Galichon P. Anaesthesia-Induced Transcriptomic Changes in the Context of Renal Ischemia Uncovered by the Use of a Novel Clamping Device. Int J Mol Sci. 2021; 22:9840. 10.3390/ijms22189840 34576005 PMC8464990

[26] Kirita Y, Wu H, Uchimura K, Wilson PC, Humphreys BD. Cell profiling of mouse acute kidney injury reveals conserved cellular responses to injury. Proc Natl Acad Sci USA. 2020; 117:15874–83. 10.1073/pnas.2005477117 32571916 PMC7355049

[27] Lemos DR, McMurdo M, Karaca G, Wilflingseder J, Leaf IA, Gupta N, Miyoshi T, Susa K, Johnson BG, Soliman K, Wang G, Morizane R, Bonventre JV, Duffield JS. Interleukin-1β Activates a MYC-Dependent Metabolic Switch in Kidney Stromal Cells Necessary for Progressive Tubulointerstitial Fibrosis. J Am Soc Nephrol. 2018; 29:1690–705. 10.1681/ASN.2017121283 29739813 PMC6054344

[28] Genescà M, Sola A, Hotter G. Actin cytoskeleton derangement induces apoptosis in renal ischemia/reperfusion. Apoptosis. 2006; 11:563–71. 10.1007/s10495-006-4937-1 16528472

[29] Gerhardt LMS, Koppitch K, van Gestel J, Guo J, Cho S, Wu H, Kirita Y, Humphreys BD, McMahon AP. Lineage Tracing and Single-Nucleus Multiomics Reveal Novel Features of Adaptive and Maladaptive Repair after Acute Kidney Injury. J Am Soc Nephrol. 2023; 34:554–71. 10.1681/ASN.0000000000000057 36735940 PMC10103206

[30] Wang P, Huang Z, Peng Y, Li H, Lin T, Zhao Y, Hu Z, Zhou Z, Zhou W, Liu Y, Hou FF. Circular RNA circBNC2 inhibits epithelial cell G2-M arrest to prevent fibrotic maladaptive repair. Nat Commun. 2022; 13:6502. 10.1038/s41467-022-34287-5 36316334 PMC9622807

[31] Pei J, Tian X, Yu C, Luo J, Zhang J, Hua Y, Wei G. GPX3 and GSTT1 as biomarkers related to oxidative stress during renal ischemia reperfusion injuries and their relationship with immune infiltration. Front Immunol. 2023; 14:1136146. 10.3389/fimmu.2023.1136146 37033969 PMC10073559

[32] Jang HR, Rabb H. Immune cells in experimental acute kidney injury. Nat Rev Nephrol. 2015; 11:88–101. 10.1038/nrneph.2014.180 25331787

[33] Ko GJ, Boo CS, Jo SK, Cho WY, Kim HK. Macrophages contribute to the development of renal fibrosis following ischaemia/reperfusion-induced acute kidney injury. Nephrol Dial Transplant. 2008; 23:842–52. 10.1093/ndt/gfm694 17984109

[34] Vinuesa E, Hotter G, Jung M, Herrero-Fresneda I, Torras J, Sola A. Macrophage involvement in the kidney repair phase after ischaemia/reperfusion injury. J Pathol. 2008; 214:104–13. 10.1002/path.2259 17973244

[35] Cheng CF, Lin H. Acute kidney injury and the potential for ATF3-regulated epigenetic therapy. Toxicol Mech Methods. 2011; 21:362–6. 10.3109/15376516.2011.557876 21495874

[36] Li X, Liu P, Sun X, Ma R, Cui T, Wang T, Bai Y, Li Y, Wu X, Feng X. Analyzing the impact of ATF3 in tumorigenesis and immune cell infiltration of ovarian tumor: a bioinformatics study. Med Oncol. 2021; 38:91. 10.1007/s12032-021-01541-7 34216322

[37] James IA, Chen CL, Huang G, Zhang HY, Velten M, Besner GE. HB-EGF protects the lungs after intestinal ischemia/reperfusion injury. J Surg Res. 2010; 163:86–95. 10.1016/j.jss.2010.03.062 20599214 PMC2922487

[38] Pressly JD, Hama T, Brien SO, Regner KR, Park F. TRIP13-deficient tubular epithelial cells are susceptible to apoptosis following acute kidney injury. Sci Rep. 2017; 7:43196. 10.1038/srep43196 28256593 PMC5335694

[39] Wu H, Huang T, Ying L, Han C, Li D, Xu Y, Zhang M, Mou S, Dong Z. MiR-155 is Involved in Renal Ischemia-Reperfusion Injury via Direct Targeting of FoxO3a and Regulating Renal Tubular Cell Pyroptosis. Cell Physiol Biochem. 2016; 40:1692–705. 10.1159/000453218 28006785

[40] Zhang Y, Lv X, Fan Q, Chen F, Wan Z, Nibaruta J, Wang H, Wang X, Yuan Y, Guo W, Leng Y. miRNA155-5P participated in DDX3X targeted regulation of pyroptosis to attenuate renal ischemia/reperfusion injury. Aging (Albany NY). 2023; 15:3586–97. 10.18632/aging.204692 37142295 PMC10449305

[41] Ding C, Dou M, Wang Y, Li Y, Wang Y, Zheng J, Li X, Xue W, Ding X, Tian P. miR-124/IRE-1α affects renal ischemia/reperfusion injury by regulating endoplasmic reticulum stress in renal tubular epithelial cells. Acta Biochim Biophys Sin (Shanghai). 2020; 52:160–7. 10.1093/abbs/gmz150 31965139

