# Supplementary material for: Identification of renal ischemia reperfusion injury-characteristic genes, pathways and immunological micro-environment features through bioinformatics approaches
Source: Aging (Albany NY). 2024 Feb 6;16(3):2123–40. doi: 10.18632/aging.205471 (PMC10911371; doi:10.18632/aging.205471)
Supplement: Supplementary Figure 1 [file aging-16-205471-s001.pdf]

SUPPLEMENTARY FIGURE

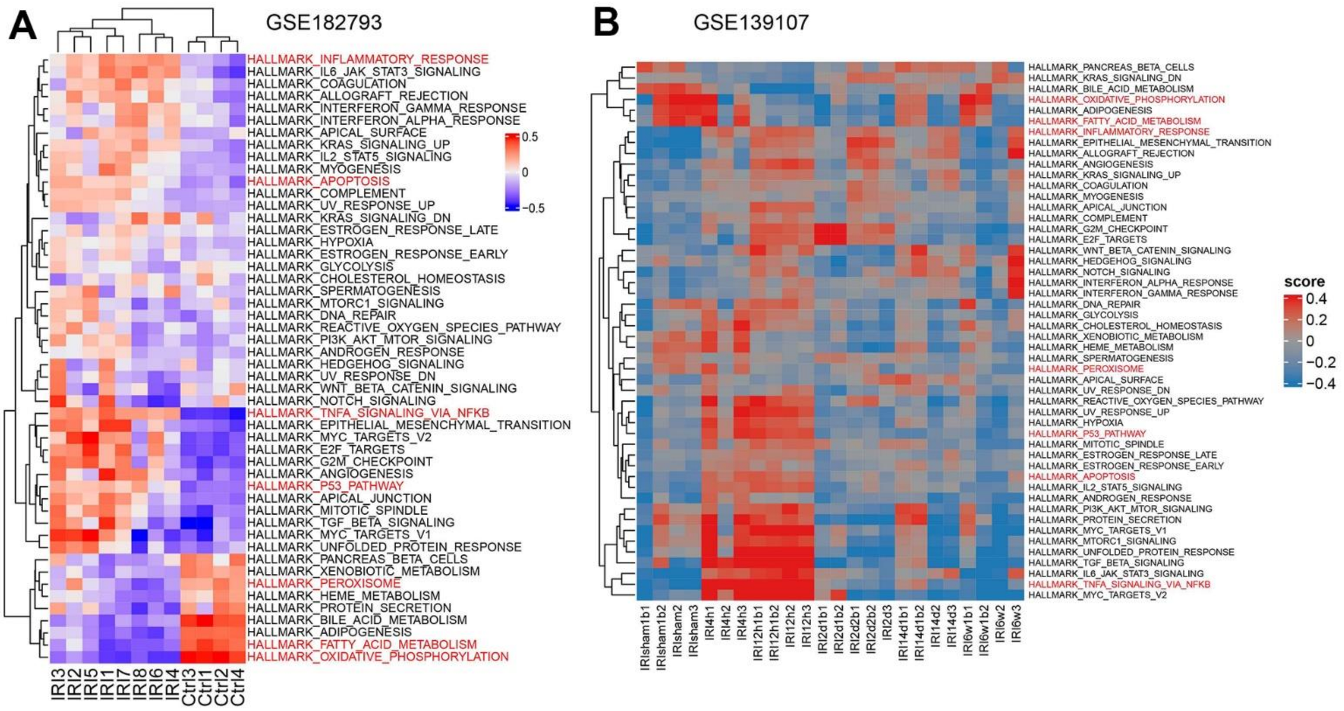

Supplementary Figure 1. GSVA analysis of external datasets. (**A**) GSVA results of GSE182793. (**B**) GSVA results of GSE139107.
